# Inhibition of neutrophil infiltration and NETs formation ameliorates neuropsychiatric and renal dysfunction in MRL/lpr mice with lupus

**DOI:** 10.1371/journal.pone.0348011

**Published:** 2026-05-22

**Authors:** Yiyao Deng, Yu Shang, Yongqiang Zhang, Delun Li, Yongle Xiong, Yan Shen, Lu Liu, Yan Ran, Pinghong He, Shuang Chen, Libo Wu, Shanshan Hu, Lulu Liu, Zhi Yang, Yan Zha, Jing Yuan

**Affiliations:** 1 Department of Nephrology, Guizhou Provincial People’s Hospital, Guiyang, Guizhou, China; 2 NHC Key Laboratory of Pulmonary Immunological Disease, Guizhou Provincial People’s Hospital, Guiyang, Guizhou, China; 3 Department of Nephrology, The First Medical Centre, Chinese PLA General Hospital, Chinese PLA Institute of Nephrology, State Key Laboratory of Kidney Diseases, National Clinical Research Centre for Kidney Diseases, Beijing Key Laboratory of Kidney Diseases, Beijing, China; 4 Guizhou University of Traditional Chinese Medicine, Guiyang, Guizhou, China; 5 The Second Clinical Medical College, Guangzhou University of Chinese Medicine, Guangzhou, China; 6 Anesthesiology department, Guizhou Provincial People’s Hospital, Guiyang, Guizhou, China; Universite Paris-Saclay, FRANCE

## Abstract

**Background:**

Patients with systemic lupus erythematosus often experience kidney and nervous system damage. It has been discovered that neutrophils and NETs play a significant role in lupus-related damage. However, whether inhibiting the infiltration of neutrophils into tissues can alleviate lupus-related brain and kidney damage remains unclear.

**Methods:**

Lupus-prone MRL/lpr mice were assigned to four groups: MRL/MpJ control, MRL/lpr model, MRL/lpr + Avacopan, and MRL/lpr + Naringenin. Cognitive functions such as memory, emotion, and learning in the mice were assessed through open field tests, Y-maze tests, and water maze tests. Changes in kidney function were also evaluated in each group. Pathological changes in the brain and kidneys of the mice, as well as the infiltration of neutrophils and the expression of NETs-related markers NE and MPO, were observed using histopathological staining, immunohistochemistry, silver staining, and multiplex immunofluorescence staining. Additionally, changes in the expression of key inflammatory factors in the brain and kidneys were examined.

**Results:**

Avacopan can improve cognitive function impairment and the progression of kidney damage in lupus-prone mice. Additionally, Avacopan has been found to reduce the infiltration of CD11b-positive and CD11b/CD16 double-positive cells into the brain and kidneys, as well as to decrease the expression of NETs-related markers NE and MPO. Notably, we observed that the main NETs marker in the kidneys of lupus mice is NE. Key inflammatory factors associated with lupus, such as IL-6, IL-17, and TNF-α, are found to be elevated in both the brain and kidney tissues of lupus mice, and Avacopan can reduce the expression of these inflammatory factors.

**Conclusion:**

Neutrophils play a significant role in systemic lupus erythematosus-related brain and kidney damage. Inhibiting the infiltration of neutrophils can mitigate the inflammatory damage and NETs-related damage caused by their infiltration. However, the detailed mechanisms by which neutrophils cause tissue damage still need further clarification. For example, it remains to be explained why there is such substantial infiltration of neutrophils into tissues, what the cell signaling mechanisms involved in damage after neutrophil infiltration are, and whether there is any impact on the regulation of other immune cells such as macrophages and T lymphocytes. Further research is needed on these aspects.

## Introduction

Systemic lupus erythematosus (SLE) is a chronic autoimmune disease characterized by widespread inflammation and multi-organ involvement, primarily affecting the kidneys and central nervous system. The prevalence of neuropsychiatric systemic lupus erythematosus (NPSLE) varies widely among studies, with estimates ranging from 37% to 95%, reflecting differences in diagnostic criteria and study populations [1–3]. Similarly, the incidence and prevalence of lupus nephritis (LN) are influenced by factors such as age, gender, race, ethnicity, geographic location, and the criteria used for diagnosis. Clinically apparent lupus nephritis can develop at any time during the course of systemic lupus erythematosus, although the risk is highest within the first 5 years after diagnosis, and many cases occur within the first 1–2 years [4–7]. Although research on the pathogenic mechanisms related to SLE has made good progress in recent years, due to the diversity of its causes, pathogenesis, and clinical manifestations, there are still many unknown mechanisms of disease that need to be elucidated.

Neutrophils play a critical role in the immune response and are increasingly recognized as key mediators in SLE pathogenesis [8–10]. While adaptive immunity, particularly the profound infiltration of double-negative T cells and CD8 + T cells, plays a dominant role in driving tissue damage in lupus nephritis and NPSLE, the innate immune system serves as a critical initiator and amplifier of this cascade. Double-negative (DN) T cells, defined as CD3 + CD4 − CD8 − T cells, are expanded in patients with systemic lupus erythematosus and are increasingly recognized as pathogenic effectors in lupus. These cells can produce IL-17 and have been detected in target organs, including the kidneys, where they may contribute to tissue inflammation and damage. In addition, accumulating evidence suggests that at least a proportion of DN T cells may originate from autoreactive CD8 + T cells after loss of CD8 expression. Therefore, the adaptive immune response, particularly DN T cells and pathogenic CD8 + T cells, should be considered an important component of lupus nephritis and neuropsychiatric lupus pathogenesis [11–13]. These cells contribute to tissue damage through mechanisms such as the formation of neutrophil extracellular traps (NETs), which are implicated in promoting autoantibody production and exacerbating organ damage [14–16]. However, it remains unclear how neutrophil infiltration into tissues and organs regulates other immune processes and induces damage mechanisms, and it is also uncertain how much benefit can be gained in the treatment of lupus by inhibiting neutrophil activity and tissue infiltration. Therefore, it is very necessary to first explore these issues through animal models.

Our previous research has shown that inhibition of neutrophil recruitment has shown potential in reducing kidney dysfunction in SLE [10]. Avacopan, a selective inhibitor of the complement 5a receptor (C5aR), has emerged as a novel agent capable of reducing neutrophil activation and tissue infiltration [17]. While Avacopan has primarily been studied in other autoimmune conditions, such as antineutrophil cytoplasmic antibody (ANCA)-associated vasculitis [18,19], its effects on SLE-related organ damage, particularly in brain and kidney dysfunction, remain underexplored.

Naringenin was included as a pharmacological comparator because it has recognized anti-inflammatory and immunomodulatory properties. Previous studies have shown that naringenin can modulate cytokine production and T-cell subsets, reduce autoantibody levels, and attenuate renal injury in lupus-prone mice. Therefore, in the present study, naringenin served as a non-C5aR-targeted comparator to help distinguish the therapeutic efficacy of Avacopan from that of a broader immunomodulatory compound [20,21].

In the current study, we employed the MRL/lpr mouse model of SLE to investigate the effects of neutrophil infiltration inhibition on brain and kidney function. Behavioral tests such as the open field test, Morris water maze, and Y-maze were conducted to assess cognitive function, while kidney function and neutrophil infiltration were evaluated to determine the extent of organ damage and inflammation. We hypothesized that inhibiting neutrophil infiltration using Avacopan would result in improvements in both brain and kidney function in SLE, offering a potential therapeutic approach to managing organ-specific complications in this disease.

## Materials and methods

### Animal model and grouping

This study employed MRL/MpJ and MRL/lpr mice, a commonly used animal model for SLE due to the MRL/lpr mice’s genetic predisposition to autoimmunity and susceptibility to lupus-like symptoms. Mice were housed in standard laboratory conditions under controlled temperature (22 ± 2°C), humidity (50 ± 10%), and a 12-hour light/dark cycle. Animals had ad libitum access to a standard rodent diet and water. All mice were acclimatized for one week prior to experimentation to reduce environmental stress. All experimental protocols and procedures were conducted following the ethical guidelines for animal experimentation. The study was reviewed and approved by the Institutional Animal Care and Use Committee at Guizhou Provincial People’s Hospital (Approval No. 2024-004). To minimize animal suffering, all invasive procedures and euthanasia were performed under deep anesthesia using isoflurane inhalation. At the end of the experiments, the mice were euthanized by cervical dislocation following deep anesthesia to ensure rapid and painless death. Every effort was made to minimize animal suffering and to reduce the number of animals used in the study.

### Drug administration

The mice were divided into four groups. Control Group: MRL/MpJ (n = 5) without any treatment. Model Group: MRL/lpr (n = 5) administered an equal dose of saline by gavage. Treatment Group: MRL/lpr + Avacopan (n = 5), 30 mg/kg by gavage, starting at 12 weeks of age, administered every other day. Avacopan was dissolved in DMSO to prepare a stock solution and then diluted with a PEG-400 and Solutol-HS-15 mixture (70:30) before gavage, with a gavage volume of 0.5 ml^10^. Naringenin Group: MRL/lpr + Naringenin (n = 5), 100 mg/kg by gavage, starting at 12 weeks of age, administered every other day. Naringenin was dissolved in DMSO to prepare a stock solution and then diluted with saline before gavage, with a gavage volume of 0.5 ml.

### Brain cognitive function assessments

Open field test: The open field test is used to evaluate general locomotion, exploration, and anxiety-like behavior in mice. Place each mouse individually in the center of a square open field apparatus, typically a 40 cm × 40 cm arena with 40 cm high walls. The test was performed in a quiet room under dim and stable illumination (~100 lux). The apparatus was cleaned with 70% ethanol between trials to eliminate olfactory cues. Allow the mouse to explore freely for 5 minutes while being recorded by a video tracking system. Assessment metrics: Locomotor activity – total distance traveled serves as a measure of overall activity and locomotion. Center vs. periphery time – The amount of time spent in the central vs. peripheral zones reflects anxiety levels; more time in the periphery often indicates anxiety-like behavior. Velocity and movement speed average speed of movement throughout the trial provides additional insights into motor function.

Y-Maze Test: The Y-Maze test assesses spatial memory and cognitive function by measuring the animal’s natural tendency to explore new environments. Procedure: Use a Y-shaped maze with three arms arranged at 120° angles. Place the mouse at the end of one arm and allow it to explore all three arms for 5 minutes. Track the order of arm entries using a video tracking system. Assessment metrics: Spontaneous alternation rate – The primary measure is the percentage of spontaneous alternation, defined as consecutive entries into each of the three arms (e.g., A → B → C without repetition). A high alternation rate reflects intact working memory. Formula: Alternation % = (Number of Alternations/ Total Entries – 2) × 100. Number of Entries – The total number of arm entries indicates general activity level, which helps differentiate memory deficits from decreased locomotion. Latency to enter arms – Time taken to enter each arm may provide additional data on cognitive exploration and motivation.

Morris Water Maze: The test assesses spatial learning and memory, often used to evaluate hippocampal-dependent cognitive function. Procedure: A circular pool filled with opaque water is used, with a hidden platform submerged 2 cm below the water surface in one of the pool quadrants. Acquisition phase: Over 5 consecutive days, mice are trained to locate the hidden platform using spatial cues around the testing room. Each trial allows a maximum of 60 seconds for the mouse to find the platform, after which it is left on the platform for 10–15 seconds. Probe trial: On the final day, the platform is removed, and the mouse is allowed to explore the pool for 60 seconds to evaluate memory retention of the platform location. Assessment metrics: Escape latency – Time taken to locate the hidden platform across trials; decreased latency over time reflects learning. Path length – Total distance traveled to reach the platform; a shorter path length over trials indicates improved spatial memory. Time in target quadrant (Probe trial) – Time spent in the quadrant where the platform was previously located, used as an indicator of memory retention. Number of platform crossings (Probe trial) – Number of times the mouse crosses the previous platform location, reflecting spatial memory accuracy. Swimming speed – Average swim speed helps rule out motor impairment as a factor affecting performance.

### Hematoxylin and Eosin (HE) staining

Brain and kidney tissues were fixed in 4% paraformaldehyde for 24 hours, embedded in paraffin, and sectioned at 5 μm thickness. Sections were deparaffinized in xylene and rehydrated through graded ethanol solutions. Staining was performed by immersing the sections in hematoxylin for 5 minutes, followed by rinsing in tap water and differentiating in 1% acid alcohol. Sections were then stained with eosin for 2 minutes, dehydrated, cleared in xylene, and mounted. Slides were observed under a light microscope to evaluate the degree of inflammatory cell infiltration, and the count of infiltrating cells was recorded for analysis. The number of immune cells infiltrating brain tissue and the number of microvessels with thrombotic occlusions are used to assess the extent of pathological damage in brain tissue. To quantify leukocyte infiltration and thrombosis, five randomly selected high-power fields (400 × magnification) were examined per tissue section using ImageJ software. Two independent, blinded observers counted the number of infiltrating cells and occluded microvessels, and the average values were calculated for statistical analysis.

### Periodic acid-Schiff (PAS) staining

Kidney tissues were fixed in 4% paraformaldehyde, embedded in paraffin, and sectioned at 5 μm thickness. After deparaffinization and rehydration, the sections were oxidized in periodic acid solution, rinsed in distilled water, and then incubated with Schiff reagent according to the manufacturer’s instructions. Sections were counterstained with hematoxylin, dehydrated, cleared, and mounted. PAS staining was used to visualize glomerular and perivascular structural alterations, including basement membrane and mesangial matrix changes, as well as overall renal tissue architecture.

### Silver staining

Silver staining was employed to detect nerve fiber damage in the brain tissue. Sections were prepared similarly as described for HE staining. Tissue sections were incubated in an ammoniacal silver solution at room temperature until staining developed, followed by rinsing in distilled water. Sections were then toned in gold chloride, fixed in sodium thiosulfate, and finally rinsed and mounted.

### Immunohistochemistry (IHC) staining

Brain sections were deparaffinized and rehydrated, followed by antigen retrieval in citrate buffer (pH 6.0) at 95°C for 15 minutes. Sections were blocked with 3% H₂O₂ for 10 minutes to quench endogenous peroxidase activity and then with 5% BSA for 30 minutes at room temperature. Primary antibodies were applied overnight at 4°C, including: MAP2 (1:2000, GB11128-2, Servicebio). After washing, sections were incubated with an HRP-conjugated secondary antibody at room temperature for 1 hour.

Signal development was performed using DAB chromogen.

### Multiplex immunofluorescence staining

Brain and kidney sections were deparaffinized, rehydrated, and subjected to antigen retrieval as in IHC. Sections were incubated with primary antibodies overnight at 4°C, including: CD11b (1:2000, GB15058, Servicebio), CD16 (1:200, GB14028, Servicebio), MAP2 (1:2000, GB11128-2, Servicebio), NE (1:8000, ET1702-78, HUABIO), MPO (1:500, GB11224, Servicebio). Sections were washed and then incubated with species-specific secondary antibodies conjugated to different fluorophores for 1 hour at room temperature. Nuclei were counterstained with DAPI, and sections were mounted with antifade mounting medium. Fluorescent images were captured using a confocal microscope, and images were analyzed for the following: Cell Counts – Number of CD11b+ and CD16 + cells in hippocampal and cerebellar regions. Fluorescence Intensity – Semiquantitative analysis of fluorescence intensity for NE and MPO as a measure of NETs formation and inflammation level.

### Inflammatory cytokine measurements

Western blot analysis was conducted to assess the protein expression levels of inflammatory cytokines in brain and kidney tissues, including IL-6, IL-17, and TNF-α. Brain and kidney tissues were homogenized in RIPA buffer (containing protease and phosphatase inhibitors) to extract total proteins. The lysates were centrifuged at 12,000 × g for 15 minutes at 4°C to remove cell debris. Supernatants were collected, and protein concentrations were determined using the BCA protein assay kit. Equal amounts of protein (40 µg per lane) were loaded onto 10−12% SDS-PAGE gels. Gels were electrophoresed at 100V for 90 minutes and then transferred to PVDF membranes at 300mA for 1.5 hours. The membranes were blocked with 5% non-fat dry milk in TBST (Tris-buffered saline with 0.1% Tween-20) for 1 hour at room temperature to prevent nonspecific binding. Membranes were incubated with the following primary antibodies overnight at 4°C: IL-6 (1:5000, GB11117-100, Servicebio), IL-17 (1:5000, GB11110-1-100, Servicebio), TNF-α (1:5000, GB115726-100, Servicebio), β-Actin (1:5000, GB15003, Servicebio). After washing the membranes with TBST three times (10 minutes each), membranes were incubated with HRP-conjugated secondary antibodies (1:3000, GB23303, Servicebio) for 1 hour at room temperature. Blots were washed again with TBST and then developed using enhanced chemiluminescence (ECL) solution. Membranes were imaged using a chemiluminescence imaging system. Western blot images were imported into ImageJ software for densitometric analysis. For each band, the area of interest was selected, and the intensity of each cytokine band was measured. The intensity of each cytokine band was normalized to the corresponding β-actin band. The relative protein expression levels were calculated as a ratio of cytokine band intensity to β-actin and expressed as a fold-change compared to the control group. Data were exported for statistical analysis to compare cytokine expression levels across groups.

### Statistical analysis

All data were collected from five mice per group. Values were reported as mean ± standard deviation (SD) and visually inspected for normality and homogeneity of variances to determine appropriate statistical methods. Before hypothesis testing, the Shapiro-Wilk test was used to evaluate data normality, and Levene’s test assessed homogeneity of variances. Normally distributed data with homogenous variances proceeded with parametric tests; otherwise, non-parametric methods were applied. One-Way ANOVA was used to analyze differences across the four groups (Control, MRL/lpr, MRL/lpr + Avacopan, and Naringenin) for each variable, including brain and kidney immune cell counts, thromboembolism counts, behavioral test data, and Western blot data. Significant ANOVA results were followed by Tukey’s post-hoc test for pairwise comparisons. Results were considered statistically significant at *P* < 0.05.

## Results

### Avacopan improves cognitive function in lupus mice

#### Open field test.

**Fig 1:**
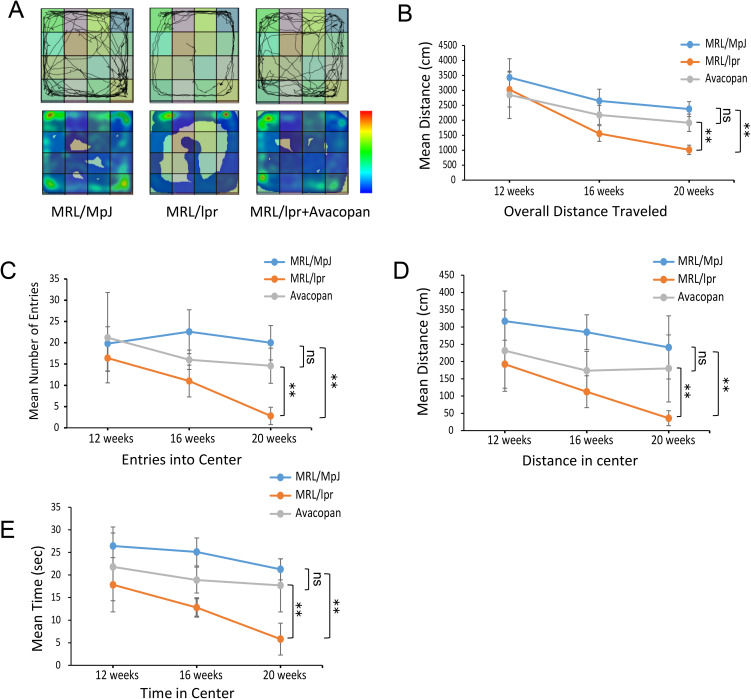
Open field test. **A)** Trajectories of mice in the open field at week 20. **B)** Total distance traveled in the open field by MRL/MpJ, MRL/lpr, and MRL/lpr+Avacopan groups at weeks 12, 16, and 20. **C)** Number of entries into the central area of the open field by MRL/MpJ, MRL/lpr, and MRL/lpr+Avacopan groups at weeks 12, 16, and 20. **D)** Total distance traveled in the central area of the open field by MRL/MpJ, MRL/lpr, and MRL/lpr+Avacopan groups at weeks 12, 16, and 20. **E)** Total time spent in the central area of the open field by MRL/MpJ, MRL/lpr, and MRL/lpr+Avacopan groups at weeks 12, 16, and 20. *p < 0.05, **p < 0.01.

The open field test was conducted at weeks 12, 16, and 20 to evaluate the general locomotor activity and exploratory behavior of MRL/MpJ, MRL/lpr, and MRL/lpr + Avacopan groups. The activity trajectories of mice at week 20 revealed that MRL/MpJ mice explored both the central and peripheral areas of the open field, while the MRL/lpr mice showed a restricted activity pattern primarily around the periphery. After Avacopan treatment, the MRL/lpr mice exhibited activity in both the peripheral and central areas of the open field (Fig 1A). The total distance traveled in the open field was measured at weeks 12, 16, and 20. As shown, all groups displayed a gradual decline in total activity distance with increasing age. At week 20, the total activity distance of the MRL/lpr group was significantly lower than that of the MRL/MpJ and MRL/lpr + Avacopan groups (Fig 1B). The frequency of entries into the central zone was observed at weeks 12, 16, and 20. It was found that both MRL/lpr and MRL/lpr + Avacopan groups showed a gradual decline in the number of entries into the central zone with increasing age, while MRL/MpJ mice exhibited no significant changes. At week 20, the MRL/lpr group had significantly fewer entries into the central zone compared to the MRL/MpJ and MRL/lpr + Avacopan groups (Fig 1C). The total distance traveled in the central zone was also measured at weeks 12, 16, and 20. A similar pattern was observed, with all groups showing a decrease in distance traveled in the central zone with increasing age. At week 20, the MRL/lpr group traveled significantly less in the central zone compared to both MRL/MpJ and MRL/lpr + Avacopan groups (Fig 1D). The total time spent in the central zone was recorded at weeks 12, 16, and 20. All groups showed a decrease in time spent in the central zone with increasing age. At week 20, the MRL/lpr group spent significantly less time in the central zone compared to the MRL/MpJ and MRL/lpr + Avacopan groups (Fig 1E).

#### Y-maze test.

**Fig 2:**
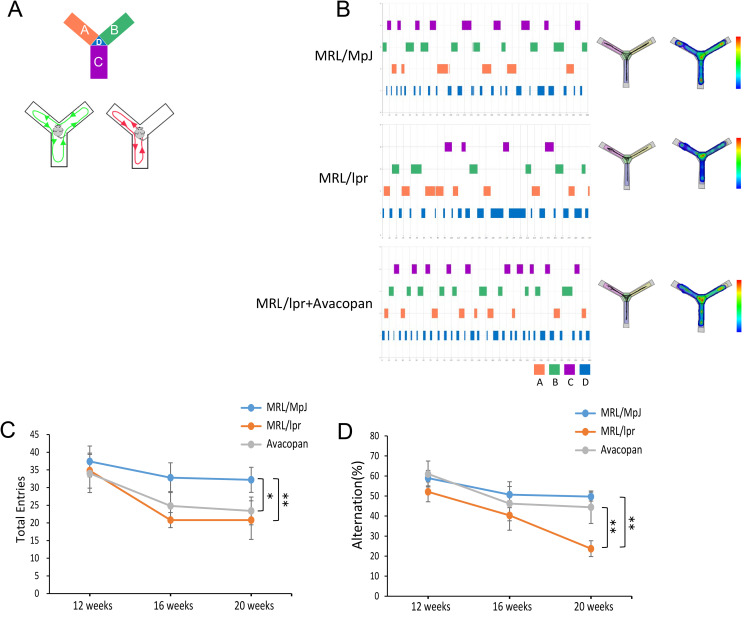
Y-maze test. **A)** Schematic diagram of the Y-maze used to explain the calculation of the alternation rate, where an alternation is counted each time a mouse sequentially traverses all three arms of the Y-maze. **B)** Alternation data, activity trajectories, and thermal imaging in the Y-maze for each group of mice at week 20. Arms A, B, and C represent the three arms of the Y-maze, and area D is the placement area for the mice. **C)** Total number of entries into each arm of the Y-maze by MRL/MpJ, MRL/lpr, and MRL/lpr+Avacopan groups observed at weeks 12, 16, and 20. **D)** Changes in alternation rate (Alternation rate) in the Y-maze for MRL/MpJ, MRL/lpr, and MRL/lpr+Avacopan groups observed at weeks 12, 16, and 20. *p < 0.05, **p < 0.01.

Cognitive flexibility and working memory were assessed using the Y-maze alternation rate and arm entries. Mice were required to traverse each arm of the Y-maze sequentially in order to complete one alternation (Fig 2A). We recorded the alternation behavior and activity trajectories, as well as heatmaps, of mice from different groups in the Y-maze experiment. Arms A, B, and C represent the three arms of the Y-maze, and the placement area for the mice is labeled as D (Fig 2B). At weeks 12, 16, and 20, we observed the total number of arm entries for MRL/MpJ, MRL/lpr, and MRL/lpr + Avacopan mice in the Y-maze experiment. It was found that as age increased, the total number of arm entries for all groups of mice gradually decreased. By week 20, the total number of arm entries for both the MRL/lpr and MRL/lpr + Avacopan mice was significantly lower compared to the MRL/MpJ group. Furthermore, no significant difference in total entries was observed between the MRL/lpr and MRL/lpr + Avacopan groups (Fig 2C). Similarly, the alternation rate in the Y-maze experiment for MRL/MpJ, MRL/lpr, and MRL/lpr + Avacopan groups was assessed at weeks 12, 16, and 20. It was observed that as age increased, the alternation rate for all groups decreased to some extent. By week 20, the alternation rate of MRL/lpr mice was significantly lower than that of MRL/MpJ and MRL/lpr + Avacopan groups (Fig 2D).

#### Water maze test.

**Fig 3:**
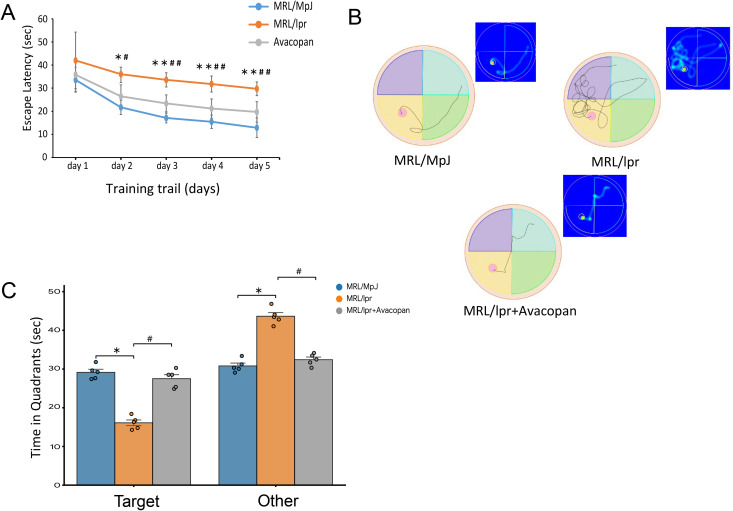
Morris water maze test. The experiment was conducted in mice at week 20 and consisted of a visible platform phase (2 days), a hidden platform training phase (3 days), and a probe test phase (1 day). **A)** Escape latency for each group of mice recorded daily during the experiment. **B)** Activity trajectories and thermal imaging of each group of mice in the water maze on day 5. **C)** Time spent by each group of mice in the platform quadrant and other quadrants; Statistical significance is indicated as follows: * *p* < 0.05, ** *p* < 0.01 compared to the MRL/MpJ group; # *p* < 0.05, ## *p* < 0.01 compared to the MRL/lpr+Avacopan group.

The water maze experiment was used to assess the spatial learning, memory ability, and navigation sense of the experimental animals. The experiment was conducted at week 20 in mice, consisting of three phases: the visible platform phase (2 days), the hidden platform training phase (3 days), and the probe test phase (1 day). During the experimental period, the escape latency for each group of mice was recorded daily. It was found that from day 2 to day 5, the escape latency of MRL/lpr mice was significantly longer than that of MRL/MpJ and MRL/lpr + Avacopan mice (Fig 3A). The activity trajectories and heatmaps of each group of mice on day 5 were recorded in the water maze. It was observed that MRL/lpr mice had more difficulty locating the platform compared to MRL/MpJ and MRL/lpr + Avacopan mice (Fig 3B). The time spent by each group of mice in the platform quadrant and in other quadrants was also recorded. It was found that MRL/lpr mice spent significantly less time in the platform quadrant compared to MRL/MpJ and MRL/lpr + Avacopan mice, while they spent significantly more time in other quadrants (Fig 3C).

### Neutrophils and NETs are associated with cognitive impairment in lupus mice

**Fig 4:**
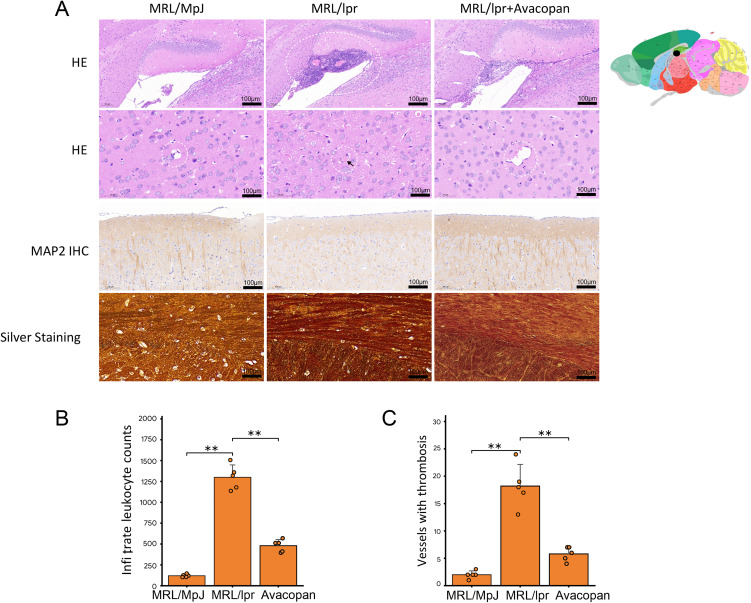
Brain histology. **A)** HE pathology staining, MAP2 immunohistochemistry, and silver staining of brain tissue from various groups of mice. The right side of panel A shows a schematic of the observed brain damage areas (Image credit: Allen Institute for Brain Science), with black dots indicating the damaged areas, primarily in the hippocampal region. **B)** Statistical count of infiltrated immune cells and microvessels affected by thromboembolic events in each group of mice. *p < 0.05, **p < 0.01.

**Fig 5:**
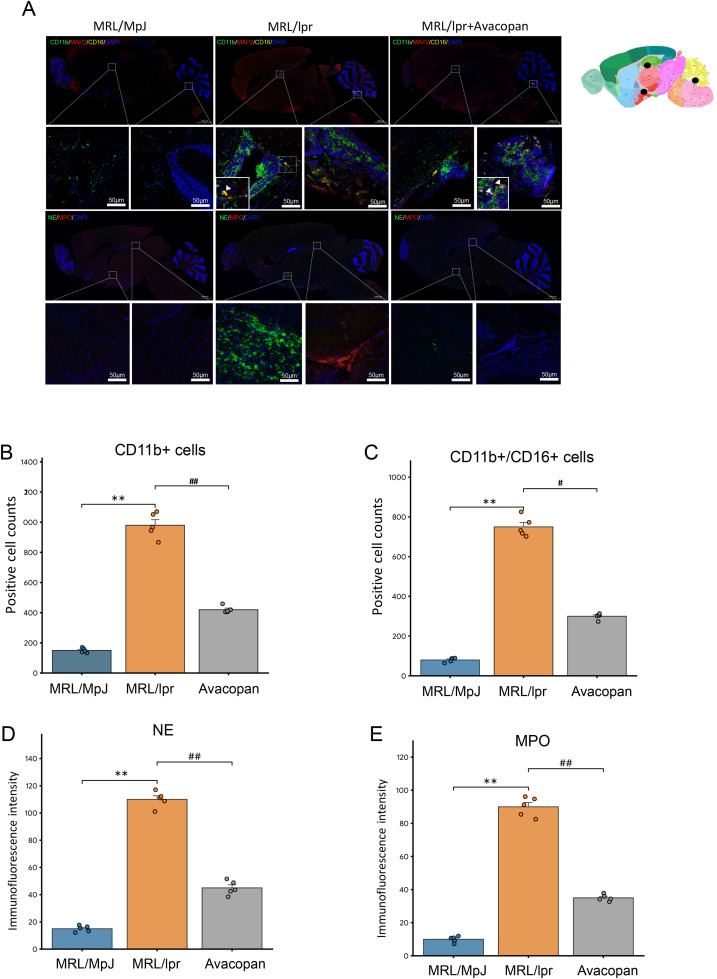
Neutrophil infiltration and NETs markers in brain tissue. **A)** Upper half: Multiplex immunofluorescence staining of CD11b, CD16, and MAP2 in brain sections from various groups of mice, with localized positive areas enlarged; white arrowheads indicate CD11b/CD16 double-positive cells. Lower half: Multiplex immunofluorescence staining of NE and MPO, with localized positive areas enlarged. The brain region schematic used as a reference is derived from the Allen Brain Atlas. **B)** and **C)** Statistical count of CD11b positive and CD11b/CD16 double-positive cells across different groups. **D)** and **E)** Semi-quantitative analysis of fluorescence intensity for NE and MPO in different groups. Statistical significance is indicated as follows: * *p* < 0.05, ** *p* < 0.01 compared to the MRL/MpJ group; # *p* < 0.05, ## *p* < 0.01 compared to the MRL/lpr+Avacopan group.

HE staining of mouse brain tissue revealed extensive infiltration of immune cells around the microvessels in the hippocampal region of MRL/lpr mice (Fig 4A). Statistical analysis of the infiltrated immune cells and the microvessels affected by thromboembolic events indicated that, at week 20, the number of infiltrated immune cells and thromboembolic microvessels in MRL/lpr mice were significantly higher than those in the MRL/MpJ and MRL/lpr + Avacopan groups (Fig 4B and 4C). MAP2 immunohistochemical staining showed that the number of subcortical neural fibers in MRL/lpr mice was significantly lower compared to MRL/MpJ and MRL/lpr + Avacopan groups (Fig 4A). Additionally, silver staining revealed that silver precipitate in the brain tissue of MRL/lpr mice was significantly higher than in the MRL/MpJ and MRL/lpr + Avacopan groups (Fig 4A). Multiplex immunofluorescence staining of brain tissues for CD11b, CD16, MAP2, NE, and MPO was consistent with the above pathological staining results, showing extensive infiltration of CD11b positive and CD11b/CD16 double positive cells in the hippocampus and cerebellum areas of MRL/lpr mice, as well as expression of NETs markers NE and MPO in the hippocampal and thalamic regions (Fig 5A). Semi-quantitative analysis of the fluorescence intensity for CD11b positive, CD11b/CD16 double positive cells, and NE and MPO indicated that the counts of CD11b/CD16 double positive cells and the fluorescence intensity of NE and MPO in MRL/lpr mouse brain tissue were significantly higher than those in the MRL/MpJ and MRL/lpr + Avacopan groups (Fig 5B, 5C, 5D and 5E).

### Avacopan improves renal function in lupus mice by inhibiting neutrophil infiltration and NETs

**Fig 6:**
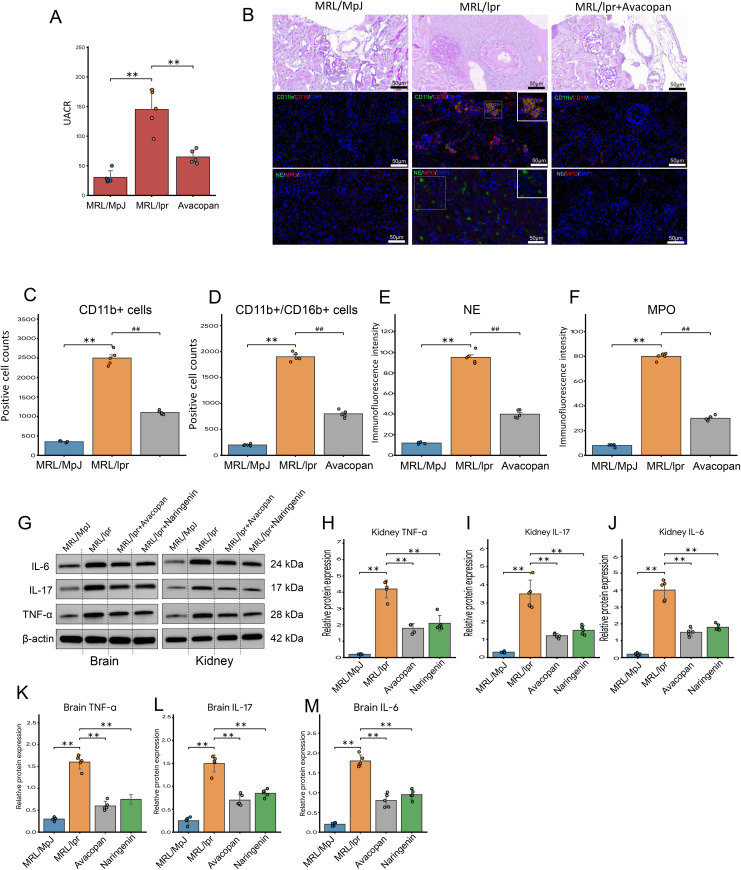
Kidney analysis and inflammatory cytokine expression. **A)** Kidney function assessment of various mouse groups at week 20, evaluated by measuring the urine albumin-to-creatinine ratio. **B)** Upper half: PAS staining of kidney tissue was used to visualize renal tissue architecture and structural injury. Immune cell infiltration was assessed by multiplex immunofluorescence staining for CD11b and CD16; lower half: Multiplex immunofluorescence staining for CD11b, CD16, NE, MPO with localized positive areas enlarged. **C)** and **D)** Statistical count of CD11b positive and CD11b/CD16 double-positive cells across different groups. **E)** and **F)** Semi-quantitative analysis of fluorescence intensity for NE and MPO in different groups; * indicates comparisons of NE fluorescence intensity, # indicates comparisons of MPO fluorescence intensity. * *p* < 0.05, ** *p* < 0.01 compared to the MRL/MpJ group; # *p* < 0.05, ## *p* < 0.01 compared to the MRL/lpr+Avacopan group. **G)** Western blot bands for IL-6, IL-17, TNF-α, and β-actin in different groups. **H** and **I)** Semi-quantitative analysis of Western blot results for brain and kidney tissues respectively; *p < 0.05, **p < 0.01.

We assessed the renal function of each group of mice along with the kidney tissue PAS pathology staining, multiplex immunofluorescence staining for CD11b, CD16, NE, MPO, and the expression of inflammatory factors IL-6, IL-17, TNF-α. At week 20, renal function tests revealed that the urine albumin-to-creatinine ratio in MRL/lpr mice was significantly higher than in the MRL/MpJ and MRL/lpr + Avacopan groups (Fig 6A). PAS staining of kidney tissue revealed marked renal structural abnormalities in MRL/lpr mice, including altered tissue architecture and crescentic lesions around small vessels. Immune cell infiltration was further evaluated by multiplex immunofluorescence staining for CD11b and CD16, which demonstrated increased inflammatory cell accumulation in the kidneys of MRL/lpr mice (Fig 6B). Semi-quantitative analysis of CD11b/CD16 double positive cells and the fluorescence intensity of NE and MPO indicated that these were significantly higher in the MRL/lpr group compared to the MRL/MpJ and MRL/lpr + Avacopan groups (Fig 6C, 6D, 6E and 6F). Western blot analysis was conducted to measure the expression of IL-6, IL-17, and TNF-α in the brain and kidney tissues at week 20. An additional group treated with naringenin, a flavonoid known for its immunomodulatory effects, served as a drug control. The expression levels of IL-6, IL-17, and TNF-α in the brain and kidney tissues of MRL/lpr mice were found to be significantly higher than those in the MRL/MpJ group, the MRL/lpr + Avacopan group, and the MRL/lpr + Naringenin group (Fig 6G, 6H, and 6I).

## Discussion

SLE is a complex, chronic autoimmune disease characterized by widespread inflammation and multisystem involvement, including significant damage to the central nervous system (CNS) and kidneys. Despite advances in understanding the pathophysiology of SLE, the mechanisms underlying the neuropsychiatric manifestations and renal damage remain incompletely understood. Current research on the roles of neutrophils and NETs in brain damage associated with SLE is limited, particularly in terms of the underlying mechanisms of injury. Our study provides a comprehensive assessment of cognitive dysfunction in a lupus mouse model, observing the involvement of neutrophils and NETs in SLE-related brain damage. By intervening in the infiltration of neutrophils, we have confirmed the significant roles of neutrophils and NETs in this context.

Neutrophils play a critical role in the pathogenesis of SLE, contributing to organ damage through multiple mechanisms, including the formation of NETs. In SLE, NETs have been implicated in the generation of autoantibodies and the amplification of inflammatory responses, contributing to both kidney and brain dysfunction. In the brain, the neuropsychiatric manifestations of lupus include cognitive dysfunction, which is commonly observed in SLE patients. MRL/lpr lupus mice exhibit brain pathology, including immune cell infiltration, endothelial damage, and neuronal degeneration [22,23]. Our study shows that Avacopan significantly reduces neutrophil infiltration in the hippocampus and cerebellum, leading to a marked improvement in cognitive function, as assessed by the open field, Y-maze, and water maze tests. The inhibition of NET formation in the brain through Avacopan treatment was further supported by the reduction of NET markers (NE and MPO) in the hippocampus, indicating a potential mechanism by which neutrophil activation contributes to neurocognitive decline in SLE.

In our study, CD11b was used as a marker for neutrophils, and we observed a large number of CD11b-expressing cells in the brain tissue of MRL/lpr mice. However, previous studies have shown that CD11b-positive brain cells are not exclusively neutrophils; they may also include activated microglia [24–26]. Therefore, it is possible that some of the CD11b-positive cells identified in our fluorescence staining results represent activated microglia, which requires further validation. Nevertheless, based on the brain pathology findings and NETs staining results, we can confirm that the majority of CD11b-positive cells in the brain tissue of MRL/lpr mice are neutrophils.

Similar pathological changes were observed in the kidneys of lupus mice. Neutrophils infiltrate the renal interstitium, where they release NETs and pro-inflammatory cytokines, leading to tissue damage and fibrosis. The MRL/lpr mice in our study exhibited significant immune cell infiltration and impaired renal function, which were alleviated by Avacopan treatment. The reduction of neutrophil infiltration and NETs in the kidney, coupled with improved renal function, highlights the potential of targeting neutrophil-mediated inflammation as a therapeutic strategy for lupus nephritis.

Inflammatory cytokines such as IL-6, IL-17, and TNF-α play pivotal roles in neutrophil activation and the progression of autoimmune diseases, including SLE [27–29]. IL-6 is a key pro-inflammatory cytokine involved in the differentiation of Th17 cells, which in turn, contribute to the activation and recruitment of neutrophils. IL-17 is another potent cytokine that promotes neutrophil survival and recruitment to sites of inflammation. TNF-α is known to exacerbate tissue damage by promoting the release of reactive oxygen species (ROS) and matrix metalloproteinases (MMPs), which degrade the extracellular matrix and enhance tissue fibrosis. In our study, we observed that the levels of IL-6, IL-17, and TNF-α were significantly elevated in the brains and kidneys of MRL/lpr mice, correlating with increased neutrophil infiltration and organ damage. Avacopan treatment effectively reduced the expression of these cytokines in both tissues, suggesting that inhibition of neutrophil infiltration also modulates the inflammatory milieu, thereby attenuating tissue injury. This finding is consistent with previous studies that have shown that neutrophils and the cytokine network they orchestrate are critical drivers of tissue damage in SLE.

Avacopan is a selective antagonist of C5aR, a key mediator of neutrophil activation and chemotaxis. C5a, a potent pro-inflammatory peptide generated during complement activation, binds to C5aR on neutrophils and other immune cells, triggering the release of inflammatory mediators and the formation of NETs [30,31]. Inhibition of C5aR with Avacopan has shown promise in clinical trials for diseases such as ANCA-associated vasculitis and lupus nephritis. In the context of lupus nephritis, Avacopan has demonstrated efficacy in reducing proteinuria and improving kidney function by modulating the immune response and reducing neutrophil-mediated damage [10,19]. In our study, Avacopan similarly improved both brain and kidney function in lupus mice, highlighting its potential as a therapeutic agent for managing SLE-related organ damage. Notably, the reduction in neutrophil infiltration, NET formation, and inflammatory cytokine expression underscores the multifaceted role of neutrophils in SLE pathogenesis and the potential of Avacopan to target these pathways effectively.

It is important to acknowledge the complexity of the immune microenvironment in MRL/lpr mice. CD11b is a pan-myeloid marker expressed not only on neutrophils but also on macrophages and microglia. To ensure specificity, our study utilized CD11b/CD16 double-positivity to distinctively quantify neutrophil infiltration. Furthermore, while Avacopan is a targeted C5aR inhibitor directly affecting neutrophil chemotaxis and NETosis, its therapeutic efficacy likely extends beyond isolated neutrophil inhibition. In particular, DN T cells (CD3 + CD4 − CD8−) are known to expand in SLE, produce IL-17, infiltrate the kidneys, and may arise from autoreactive CD8 + T cells, further supporting the importance of adaptive immune dysregulation in lupus target-organ injury. We hypothesize that by blocking the C5aR axis and reducing NET release, Avacopan indirectly attenuates the exposure of autoantigens and the inflammatory milieu, thereby subsequently mitigating the broader cascade of T cell and general immune infiltration. Future studies utilizing neutrophil-specific C5aR knockout models or single-cell transcriptomics will be necessary to precisely dissect the specific versus general immunosuppressive effects of Avacopan in SLE.

Recent studies have highlighted the multifaceted role of neutrophils in influencing other immune cells once they infiltrate tissues and organs. Beyond their well-known role in acute inflammation and pathogen clearance, neutrophils actively interact with various immune cells, shaping the immune microenvironment. For instance, neutrophils can recruit and activate macrophages by releasing cytokines such as IL-1β and TNF-α, enhancing the inflammatory response and promoting tissue damage [32,33]. Additionally, neutrophil-derived NETs not only damage endothelial cells but also provide a scaffold for antigen presentation, potentially activating dendritic cells and perpetuating T-cell responses [34,35]. In lupus and other autoimmune diseases, neutrophils have been shown to contribute to the activation of T cells. For example, IL-6 and IL-17 produced in neutrophil-enriched environments can promote the differentiation of Th17 cells, which are implicated in autoimmune pathology [36–38]. Moreover, neutrophils can interact with B cells through the release of BAFF, thereby enhancing autoantibody production and contributing to systemic autoimmunity [32]. The infiltration of neutrophils into tissues like the brain and kidneys, as seen in our study, may also exacerbate local immune dysregulation. In the brain, neutrophil-induced activation of microglia and astrocytes can amplify neuroinflammation, potentially contributing to neuropsychiatric manifestations of lupus. In the kidneys, neutrophils can influence the activation of resident macrophages and promote the deposition of immune complexes, accelerating the progression of lupus nephritis. These interactions underscore the complex and bidirectional crosstalk between neutrophils and other immune cells, which not only propagates inflammation but also disrupts tissue homeostasis. Further studies are needed to elucidate these mechanisms and explore potential therapeutic strategies targeting neutrophil-mediated immune modulation.

## Conclusion

Our study provides compelling evidence that neutrophils and NETs play a vital role in the pathogenesis of SLE-related brain and kidney dysfunction. The selective inhibition of neutrophil infiltration and NET formation with Avacopan improves cognitive function and renal health in MRL/lpr lupus mice, offering a novel therapeutic approach for SLE. The reduction of key inflammatory cytokines, including IL-6, IL-17, and TNF-α, further supports the critical role of neutrophils in driving the immune response in SLE. These findings suggest that Avacopan, through its effects on neutrophil recruitment and activation, could be a valuable addition to the therapeutic arsenal for treating SLE and its associated organ damage. Further clinical trials are needed to confirm the efficacy and safety of Avacopan in lupus patients, especially in the context of neuropsychiatric manifestations and lupus nephritis. The growing body of evidence surrounding neutrophils and NETs as therapeutic targets offers new hope for patients suffering from the devastating consequences of SLE.

## Supporting information

S1 Raw Images Original uncropped and unadjusted Western blot images corresponding to Fig 6G, including brain and kidney tissue blots for IL-6, IL-17, TNF-α, and β-actin. (PDF)

S1 Dataset Raw quantitative data supporting the analyses presented in Fig 1, including open field test parameters measured at weeks 12, 16, and 20. (XLSX)

S2 Dataset Raw quantitative data supporting the analyses presented in Fig 2, including Y-maze behavioral test results and alternation rates. (XLSX)

S3 Dataset Raw quantitative data supporting the escape latency analyses presented in Fig 3A of the Morris water maze experiment. (XLSX)

S4 Dataset Raw quantitative data supporting the platform quadrant retention analyses presented in Fig 3C of the Morris water maze experiment. (XLSX)

S5 Dataset Raw quantitative data supporting the histological analyses presented in Fig 4, including immune cell infiltration and thromboembolic microvessel quantification in brain tissues. (XLSX)

S6 Dataset Raw quantitative data supporting the immunofluorescence analyses presented in Fig 5, including CD11b-positive cells, CD11b/CD16 double-positive cells, and NETs marker fluorescence intensity in brain tissues. (XLSX)

S7 Dataset Raw quantitative data supporting the renal function and kidney immunofluorescence analyses presented in Fig 6, including UACR measurements and fluorescence quantification of CD11b, CD16, NE, and MPO in kidney tissues. (XLSX)

S8 Dataset Raw quantitative data supporting the Western blot analyses presented in Fig 6, including densitometric quantification of IL-6, IL-17, and TNF-α expression in brain and kidney tissues. (XLSX)
