# Supplementary material for: Inhibition of neutrophil infiltration and NETs formation ameliorates neuropsychiatric and renal dysfunction in MRL/lpr mice with lupus
Source: PLoS One. 2026 May 22;21(5):e0348011. doi: 10.1371/journal.pone.0348011 (PMC13196981; doi:10.1371/journal.pone.0348011)

Source Data for Figure 6G

Five biological duplication of full uncropped blots and of brain

Detection method: Chemiluminescence imaging system

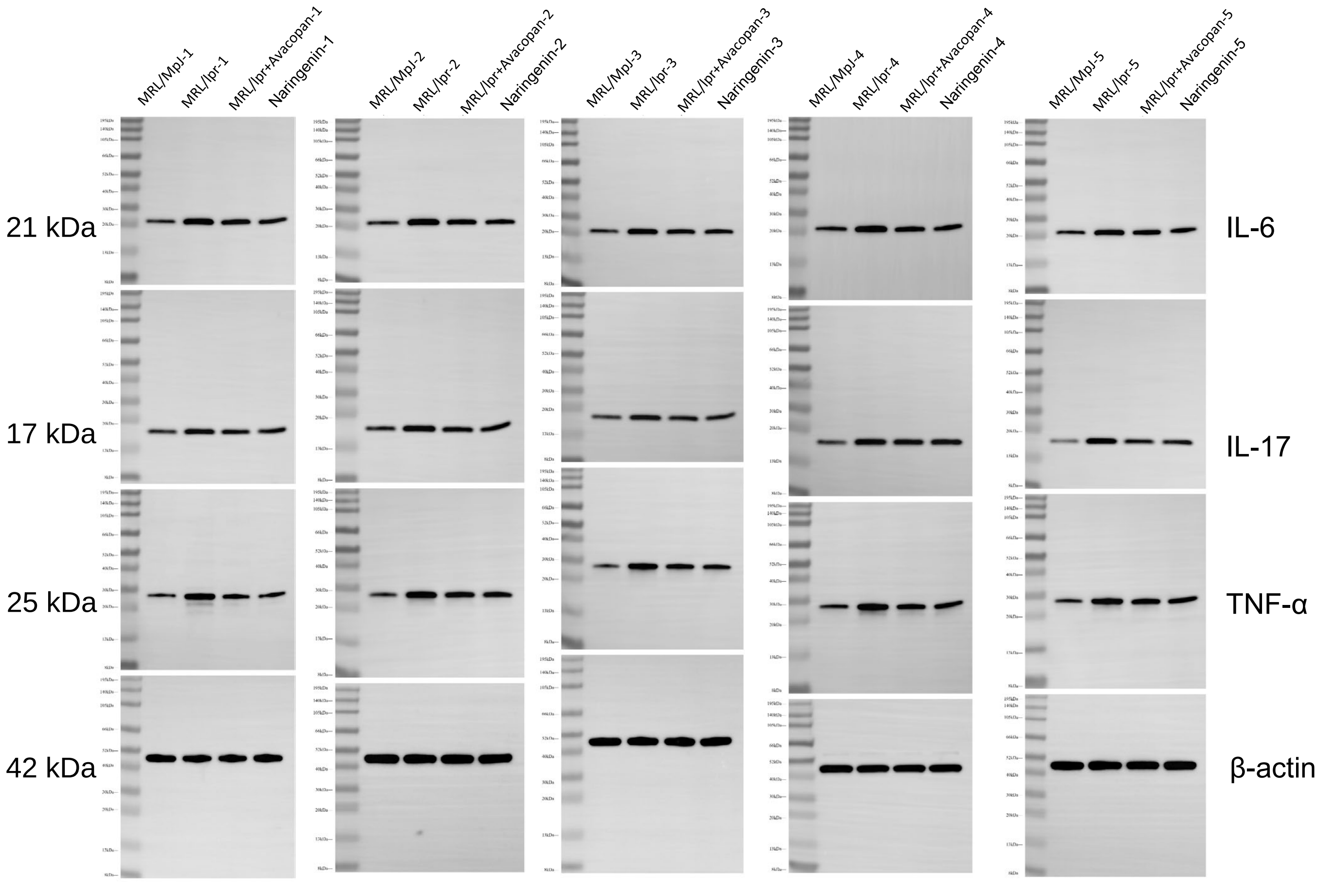

Source Data for Figure 6G

Five biological duplication of full uncropped blots and of kidney

Detection method: Chemiluminescence imaging system

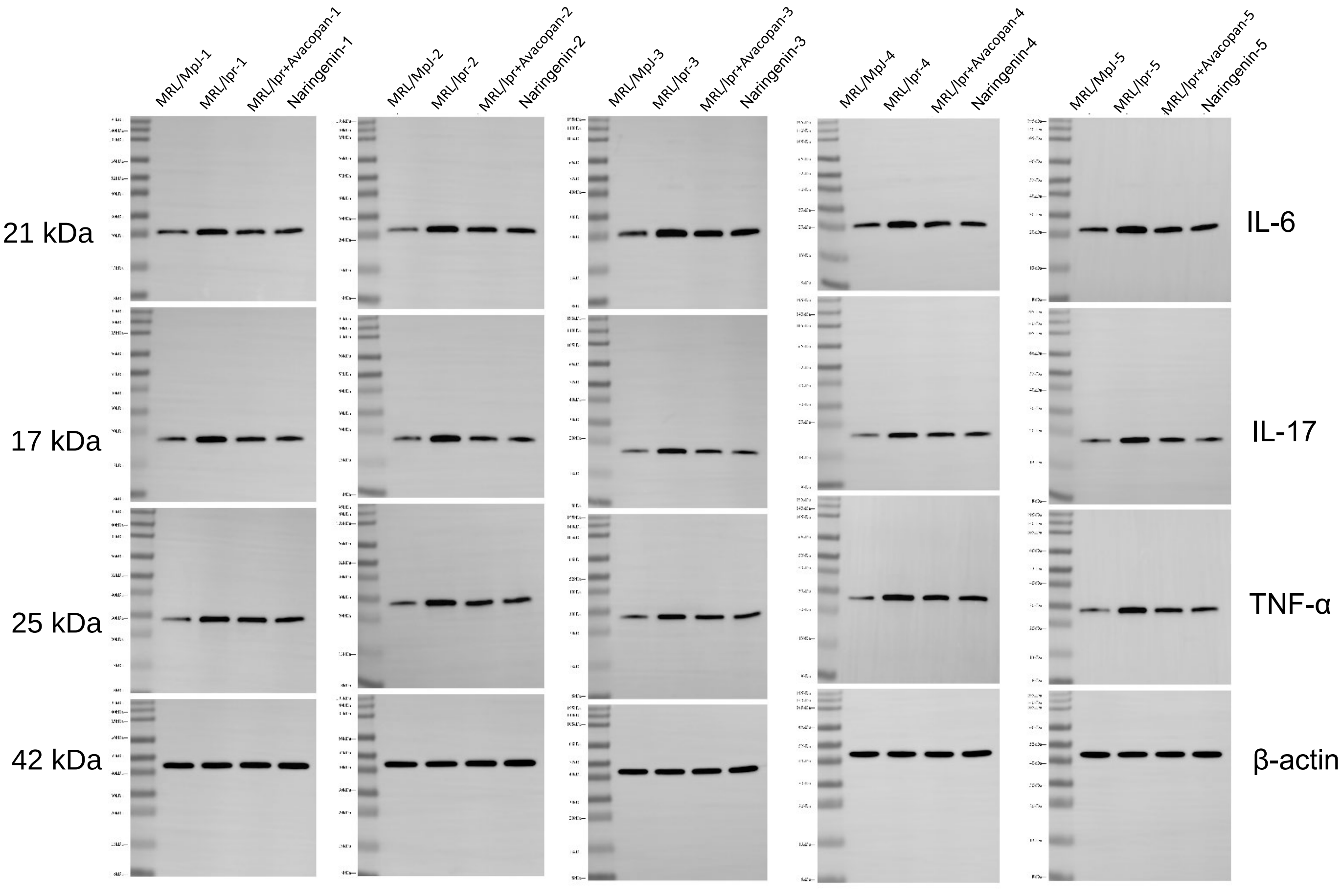

Supplement: S1 Raw Images — (PDF) [file pone.0348011.s001.pdf]
